# Effects of low molecular weight heparin on the polarization and cytokine profile of macrophages and T helper cells *in vitro*

**DOI:** 10.1038/s41598-018-22418-2

**Published:** 2018-03-08

**Authors:** Valentina Bruno, Judit Svensson-Arvelund, Marie Rubér, Göran Berg, Emilio Piccione, Maria C. Jenmalm, Jan Ernerudh

**Affiliations:** 1 Section of Gynecology and Obstetrics, Academic Department of Biomedicine and Prevention, and Clinical Department of Surgery, Tor Vergata University Hospital, Viale Oxford 81, 00133 Rome, Italy; 2 Department of Clinical and Experimental Medicine, Linköping University, SE-581 83 Linköping, Sweden; 3 Department of Obstetrics and Gynecology, and Department of Clinical and Experimental Medicine, Linköping University, SE-581 83 Linköping, Sweden; 4 Department of Clinical Immunology and Transfusion Medicine, and Department of Clinical and Experimental Medicine, Linköping University, SE-581 83 Linköping, Sweden

## Abstract

Low molecular weight heparin (LMWH) is widely used in recurrent miscarriage treatment. The anti-coagulant effects are established, while immunological effects are not fully known. Our aim was to assess LMWH effects on activation and polarization of central regulatory immune cells from healthy women, and on placenta tissues from women undergoing elective abortions. Isolated blood monocytes and T helper (Th) cells under different activation and polarizing conditions were cultured with or without LMWH. Flow cytometry showed that LMWH exposure induced increased expression of HLA-DR and CD206 in macrophages. This phenotype was associated with increased secretion of Th17-associated CCL20, and decreased secretion of CCL2 (M2-associated) and CCL22 (Th2), as measured by multiplex bead array. In accordance, LMWH exposure to Th cells reduced the proportion of CD25highFoxp3+ regulatory T-cells, intensified IFN-γ secretion and showed a tendency to increase the lymphoblast proportions. Collectively, a mainly pro-inflammatory effect was noted on two essential tolerance-promoting cells. Although the biological significancies of these *in vitro* findings are uncertain and need to be confirmed *in vivo*, they suggest the possibility that immunological effects of LMWH may be beneficial mainly at an earlier gestational age to provide an appropriate implantation process in women with recurrent miscarriage.

## Introduction

Low molecular weight heparin (LMWH) is widely used in clinical practice as an empirical therapy for recurrent miscarriages (RM)^1^. RM is a clinical condition characterized by a predisposition to break tolerance involving different auto-antibodies (such as anti-cardiolipin antibodies, anti-nuclear antibodies (ANA), and anti-thyroid-peroxidase (TPO)-antibodies), increased pro-inflammatory responses (such as high plasma levels of TNF, IFN-γ and IL-6, increased T helper (Th)-17 and decreased Foxp3^+^ T regulatory cells in peripheral blood), and a dysregulation of maternal immune response to fetal or placental trophoblast antigens^2^. The rationale behind the use of LMWH in RM is that, beyond its well-documented anticoagulant effects and inhibitory action on the complement system, LMWH potentially exerts immune-modulatory and anti-inflammatory actions, which could counteract the pro-inflammatory response that is involved in RM pathogenesis^3–9^. Furthermore, LMWH may also be involved in regulating pregnancy-promoting processes at the fetal-maternal interface, like the prevention of trophoblast apoptosis^10^, enhancement of trophoblast invasiveness^11^, improvement of the endothelial and vascular environments, and regulation of embryo implantation^12^. In regards to the immunological effects of LMWH, mainly aspects of innate immunity have been studied^13–20^, while there is limited information on mechanisms involving central immune regulatory cells at the fetal-maternal interface.

After implantation, the uterine endometrium (during pregnancy named the decidua) is infiltrated by trophoblast cells of fetal origin. In order to regulate trophoblast invasion and to promote fetal tolerance and homeostasis, the decidua holds a unique composition of immune cells with specialized properties^21^. For fetal tolerance, decidual macrophages and regulatory T (Treg) cells are of particular relevance; both cell types being enriched in the decidua and with an immune regulatory profile^22,23^. The decidual macrophages are of a regulatory M2-like phenotype^24,25^ and Treg cells^26,27^ show an augmented suppressive profile in the decidua. Accordingly, aberrant activation and polarization of both cell types may be involved in pregnancy complications including RM, as has been shown for macrophages^28^ and by an altered Treg/Th17 balance^29^. Despite the crucial role of decidual macrophages and Treg cells and the potential role of their dysregulation in RM, there is limited information on LMWH effects on these cells.

The aim of the present study was to assess the potential immunoregulatory properties of LMWH, with a particular regard to the effects on macrophages and Th cells. We therefore used *in vitro* cellular models to evaluate the direct effects of LMWH on the polarization and activation of macrophages and Th cells by characterizing their phenotype and cytokine/chemokine secretion patterns. In addition, the effects of LMWH on cytokine and chemokine secretion by 1^st^ trimester placental tissue was evaluated, since this pathway could be an indirect way of modulating macrophages and Th cells.

## Materials and Methods

### Subjects

For the *in vitro* assays with macrophages and T cells, blood samples were collected from 20 healthy non-pregnant female volunteers, between 18–45 years of age, not taking hormonal contraceptives or any other medication. For the *in vitro* assay with placental tissue, first-trimester placental tissues were collected from 10 healthy pregnant women undergoing elective surgical abortions at Linköping University Hospital (Linköping, Sweden). All pregnancies were viable, and the median gestational week was 10 (range 9–12), as determined by crown-rump length using ultrasound. Misoprostol (Cytotec; Searle) was given to all women prior to surgery. The study was approved by the regional ethical board in Linköping, and written informed consent was obtained from all subjects. All experiments were performed in accordance with the Helsinki Declaration ethical principles for medical research.

### Isolation of blood cells

Peripheral blood mononuclear cells (PBMCs) were isolated on a LymphoPrep gradient (Axis-Shield, Dundee, Scotland, UK), according to the manufacturer’s instructions, followed by washing in HBSS (Invitrogen). Isolated PBMCs were used for isolation of CD14^+^ monocytes or CD4^+^ T cells by positive selection using immunomagnetic cell sorting. PBMCs were resuspended in sterile MACS buffer (phosphate buffer saline (PBS) supplemented with 2 mM EDTA (Sigma-Aldrich, Saint Louis, Missouri, USA) and 0.5% fetal bovine serum (FBS)), and the CD14^+^ or CD4^+^ cells were isolated with anti-CD14 or anti-CD4 mAb-coated MicroBeads, according to the manufacturer’s protocol), using MS MACS columns (all from MiltenyiBiotec, BergischGladbach, Germany).

### Cell cultures of macrophages and CD4^+^ T helper cells

To analyze the effects of LMWH on Th cells and macrophages, LMWH, Innohep® (tinzaparin sodium) was added to Th cell or macrophage cultures at two different doses: 1 IU and 10 IU. These doses correspond approximately to those reached *in vivo* after low dose intra-muscular LMWH injection, commonly used in the clinical practice for RM patients. The models of macrophage and Th cell activation and polarization have been optimized and used in previous studies from our group^22,25^.

Macrophages were generated from CD14^+^ monocytes in 24-well plates, as previously described^22,25^, in the presence of 5 ng/ml recombinant human granulocyte-macrophage colony-stimulating factor (GM-CSF) or 50 ng/ml macrophage colony-stimulating factor (M-CSF) (PeproTech, Rocky Hill, United States), in the presence or absence of LMWH. Cells were cultured at a density of 500,000 cells/well in 500 µl cell culture medium for 6 d at 37 °C and 5% CO_2_. In macrophage cultivation experiments, cells from 7–8 healthy controls were used, and exposed to GM-CSF, M-CSF and LMWH in the same experiment. To analyze the effects of LMWH on resting, as well as activated, Th cells, isolated CD4^+^ T cells were either cultured unstimulated or stimulated with anti-CD3 and anti-CD28 Abs. 96-well plates (Corning Costar, Sigma-Aldrich) were pre-coated with 0.25 mg/ml anti-CD3 and anti-CD28 Abs (low endotoxin; AbDSerotec, Raleigh, North Carolina, USA) for 2 h at 37 °C, followed by washing with PBS. For unstimulated cells, plates were coated with PBS only. CD4^+^ T cells were cultured, in the presence or absence of LMWH, at a density of 50,000 cells/well in 150 µl T cell culture medium, consisting of IMDM (Invitrogen) supplemented with L-glutamine (292 mg/ml; Sigma-Aldrich), sodium bicarbonate (3.024 g/l; Sigma-Aldrich), penicillin (50 IE/ml), streptomycin (50 mg/ml) (Cambrex), 100× nonessential amino acids (10 ml/l; Invitrogen), 5% heat-inactivated FBS, for 3 d at 37 °C and 5% CO_2_. In T cell experiments, cells from 7–19 healthy controls were used (see figure legends for details).

### Placental explants

To measure the influence of LMWH on placental tissue, we measured secreted cytokines and chemokines from placental explants exposed or not exposed to LMWH *in vitro*. Immediately after collection of 1st trimester tissue, the decidua was removed, and the fetal placental tissue was processed. The fetal part of the placenta (simply called “placenta” in this paper) was rinsed with sterile saline to remove traces of maternal blood, and washed in sterile PBS. The placental villi were dissected into small pieces (∼1–2 mm in diameter) and placed in 24-well plates with culture medium consisting of RPMI 1640 (Life Technologies-Invitrogen, Burlington- BRL) supplemented with 10% heat-inactivated FBS (PAA Laboratories, Paris, France) and 1% PEST/L-glutamine (Life Technologies- Invitrogen, BRL). A total of ∼50–100 mg of wet tissue was added to each well, pre-filled with 10 µl culture medium/mg tissue. The placental explants (n = 10) were incubated for 20–24 h at 37 °C and 5% CO_2_, without or with 1 IU or 10 IU of LMWH. The conditioned medium (CM) was collected, centrifuged, and stored in aliquots at −70 °C.

### Flow cytometry staining and analysis

Cells were resuspended in PBS supplemented with 0.1% FBS (PBS 0.1% FBS) and stained with Abs for extracellular staining and their corresponding isotype controls (for Ab details, see Supplemental Table I) for 30 min at 4 °C in the dark. PBS 0.1% FBS was added, followed by centrifugation at 500 g for 5 min. The cell pellet was resuspended in PBS 0.1% FBS for final flow cytometric analysis. Alternatively, for staining with 7-aminoactinomycin D and Annexin V-PE (BD Biosciences, Franklin Lakes, New Jersey, USAs), which were used to assess viability, cells were resuspended and washed in Annexin V–binding buffer (BD Biosciences). After extracellular staining, cells were permeabilized, according to the manufacturer’s instructions, using the Foxp3 staining kit (eBioscience), followed by staining with anti-human Foxp3, T-bet, GATA-3, or ROR-γt (for Ab details, see Supplemental Table I) for 30 min at 4 °C. After washing, cells were resuspended in PBS 0.1% FBS.

### Analysis and gating strategy

Data were acquired using a FACSCanto II and analyzed with FACSDiva software version 6.1.2 (BD Biosciences) or Kaluza software version 1.1 (Beckman Coulter, Fullerton, California). Isotype controls were used to set the cut-off for macrophage markers, as well as for some of the CD4 markers (CD25 and the intra-cellular Foxp3, T-bet, GATA-3, or ROR-γt). The CD25^high^ gate was set according to a slightly lowered expression of CD4 on CD4^+^ cells (CD4^dim^)^30^. The percentage of HLA-DR– and CD69-expressing cells was set according to the unstimulated control population. The median fluorescence intensity (MFI) ratio, was calculated as the ratio between the MFI of specific Ab-stained cells and the MFI of isotype control-stained cells.

### Analysis of cytokines and chemokines with multiplex bead assay

Multiplex bead assay kits were used, according to the manufacturer’s protocols (Millipore, Merck KGaA, Darmstadt, Germany), to analyze supernatants from Th cell cultures for the following factors (detection limits are shown in brackets): GM-CSF (9.6 pg/ml), IL-2 (110 pg/ml), IL-10 (5.2 pg/ml), IL-17A (5.5 pg/ml), IFN-γ (33 pg/ml), IL-1β (7.3 pg/ml), IL-6 (4.7 pg/ml), TNF (5.2 pg/ml); and supernatants from macrophage cultures for CCL2 (98 pg/ml), CCL22 (177 pg/ml), CCL20 (10 pg/ml), CXCL1 (102 pg/ml), CXCL10 (2.6 pg/ml), IL-12 p70 (56 pg/ml), IL-23 (74 pg/ml), TNF (5.0 pg/ml) and IL-10 (4.6 pg/ml). CM from placental explants were analyzed for CCL2 (4.8 pg/ml), CCL22 (3.0 pg/ml), CCL20 (4.3 pg/ml), CXCL10 (3.2 pg/ml), CXCL1–3 (6.8 pg/ml), CXCL8 (1.8 pg/ml), GM-CSF (1.5 pg/ml), M-CSF (85 pg/ml), IL-10 (1.6 pg/ml), IL-1β (1.6 pg/ml), IL-6 (1.7 pg/ml), VEGF (16 pg/ml), TRAIL (5.1 pg/ml) and sFasL (0.04 pg/ml). The analyses were performed using the Luminex 200 IS system (Millipore) and the MasterPlex QT 2010 software (MiraiBio). Values below the detection limit were assigned half the value of the detection limit.

### Data analysis and statistics

Kolmogorov-Smirnov test was used to analyze data distribution. The majority of the flow cytometry data was normally distributed and therefore analyzed with one-way ANOVA followed by the Sidak’s post hoc test for paired data. Data from multiplex bead assays were not normally distributed and accordingly analyzed with the Friedman test followed by the Wilcoxon matched-pairs tests. Differences were considered statistically significant when the several-group comparison (ANOVA or Friedman test) was p < 0.05 and the post hoc test (Sidak or Wilcoxon) was p < 0.05, while differences were considered a tendency when the several-group test was p < 0.09 and the post hoc test was p < 0.05. The relative difference (increase or decrease) was calculated by expressing the exposed level as % of the unexposed levels. Flow cytometry data are expressed as mean and SD, whereas data from the multiplex bead assay are presented as box plots showing medians and inter-quartile ranges, while whiskers show the range. All data were analyzed using GraphPad Prism version 6.0 (La Jolla, CA, USA).

## Results

### Effects of LMWH on macrophages

**Figure 1:**
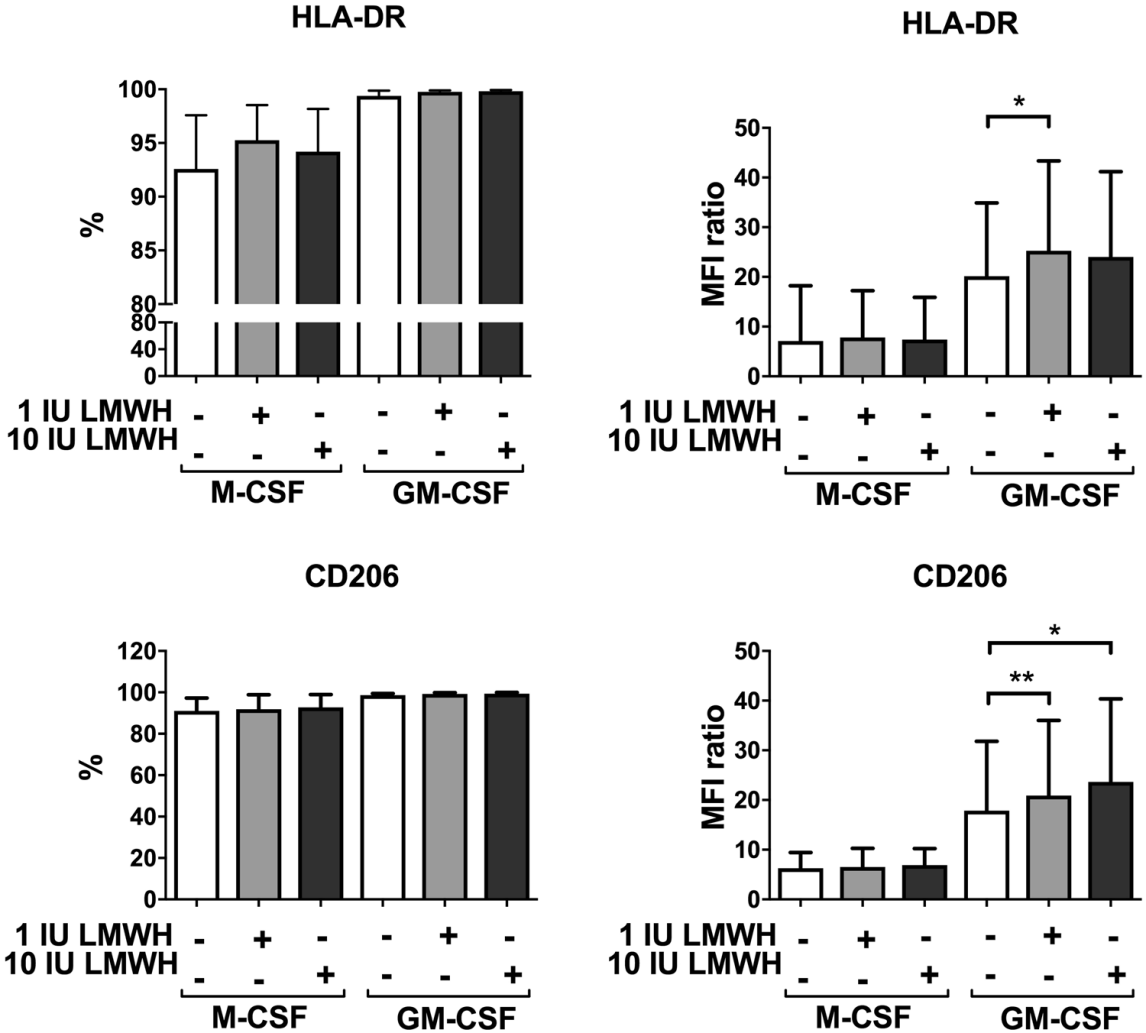
Effects of LMWH on macrophage polarization. Effect of LMWH on macrophage phenotype markers, measured as proportion of cells (%) and relative expression of the entire population (median fluorescence intensity, MFI). Macrophages were cultured for 6 days with GM-CSF or M-CSF in the absence or presence of 1 or 10 IU LMWH. Data are expressed as mean ± S.D. One way ANOVA and Sidak’ s post hoc test: *p < 0.05; **p < 0.005; n = 8.

**Figure 2:**
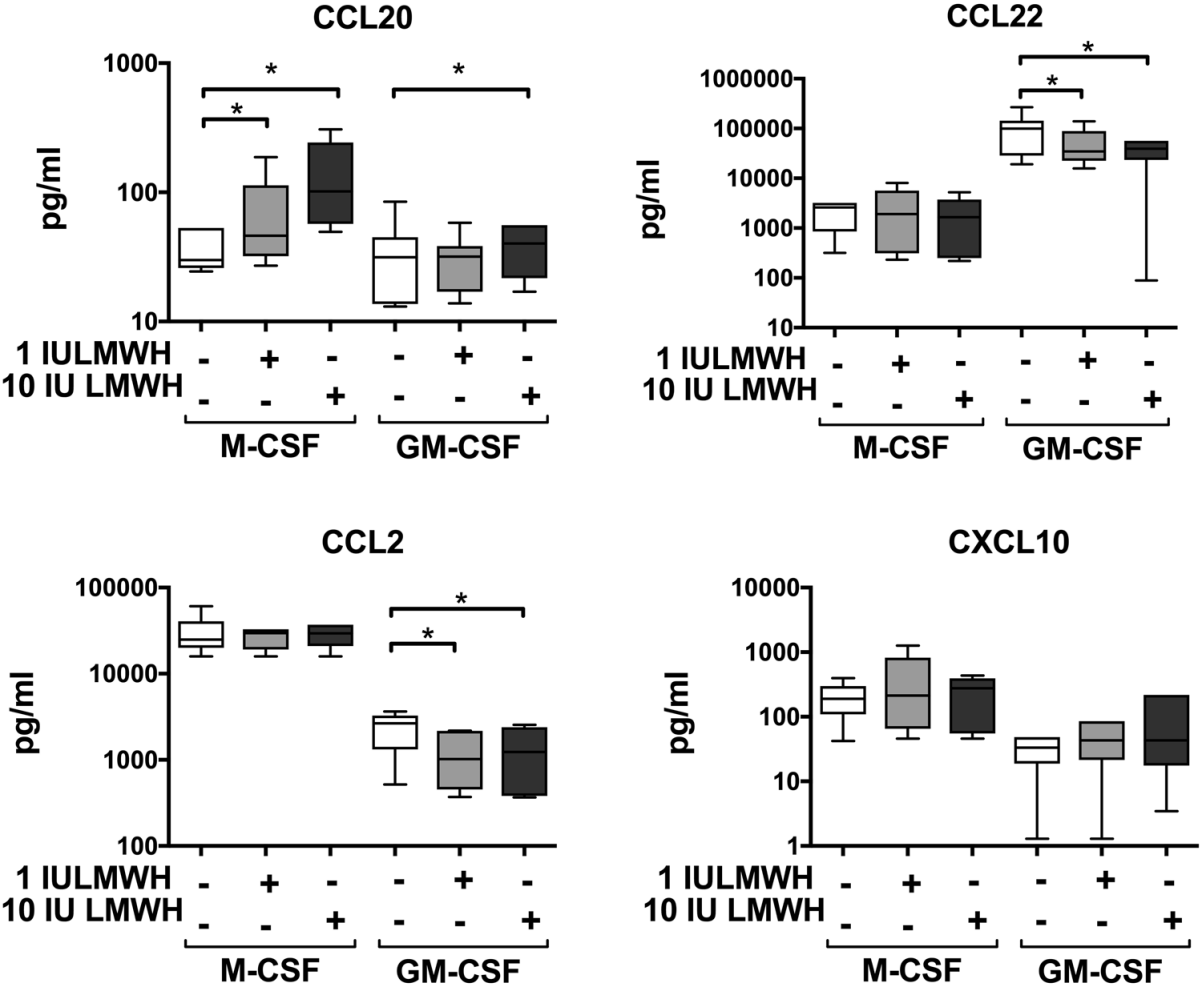
Effects of LMWH on the production of chemokines by macrophages. Macrophages were cultured with GM-CSF and M-CSF for 6 days in the absence or presence of 1 or 10 IU LMWH. Data are expressed as box plots showing median and inter-quartile range (box) and range (whiskers). When Friedman test was p < 0.05, Wilcoxon test was performed: *p < 0.05; n = 7.

To assess the effect of LMWH on macrophage polarization, CD14^+^ blood monocytes, isolated from non-pregnant women, were cultured with polarizing growth factors in the presence or absence of LMWH. GM-CSF was used to induce an M1-like phenotype (mainly pro-inflammatory), in order to asses if LMWH can reverse the M1 phenotype and increase expression of M2 phenotype markers, measured as proportion of positive cells (%) or relative expression of the entire population (median fluorescence intensity, MFI). GM-CSF-macrophages generated in the presence of LMWH acquired a phenotype characterized by a significantly higher expression of HLA-DR (MFI, a relative increase of 25%) and CD206 (MFI, 17–33% increase), compared with GM-CSF-macrophages not exposed to LMWH (Fig. 1, representative histograms are shown in Supplemental Fig. 1), while CD163 and CD209 expression was not affected by LMWH (Supplemental Fig. 2). Furthermore, LMWH exposure significantly decreased the secretion of CCL2 (53–62% decrease) and CCL22 (61–65% decrease) in GM-CSF-cultured macrophages, while it increased CCL20 production (28–240% increase) in both GM-CSF and M-CSF cultured macrophages (Fig. 2). LMWH did not affect cytokine secretion (IL-10, IL-12, IL-23 and TNF), neither in GM-CSF nor in M-CSF-cultured macrophages (Supplemental Fig. 3).

### Effects of LMWH on Th cells

**Figure 3:**
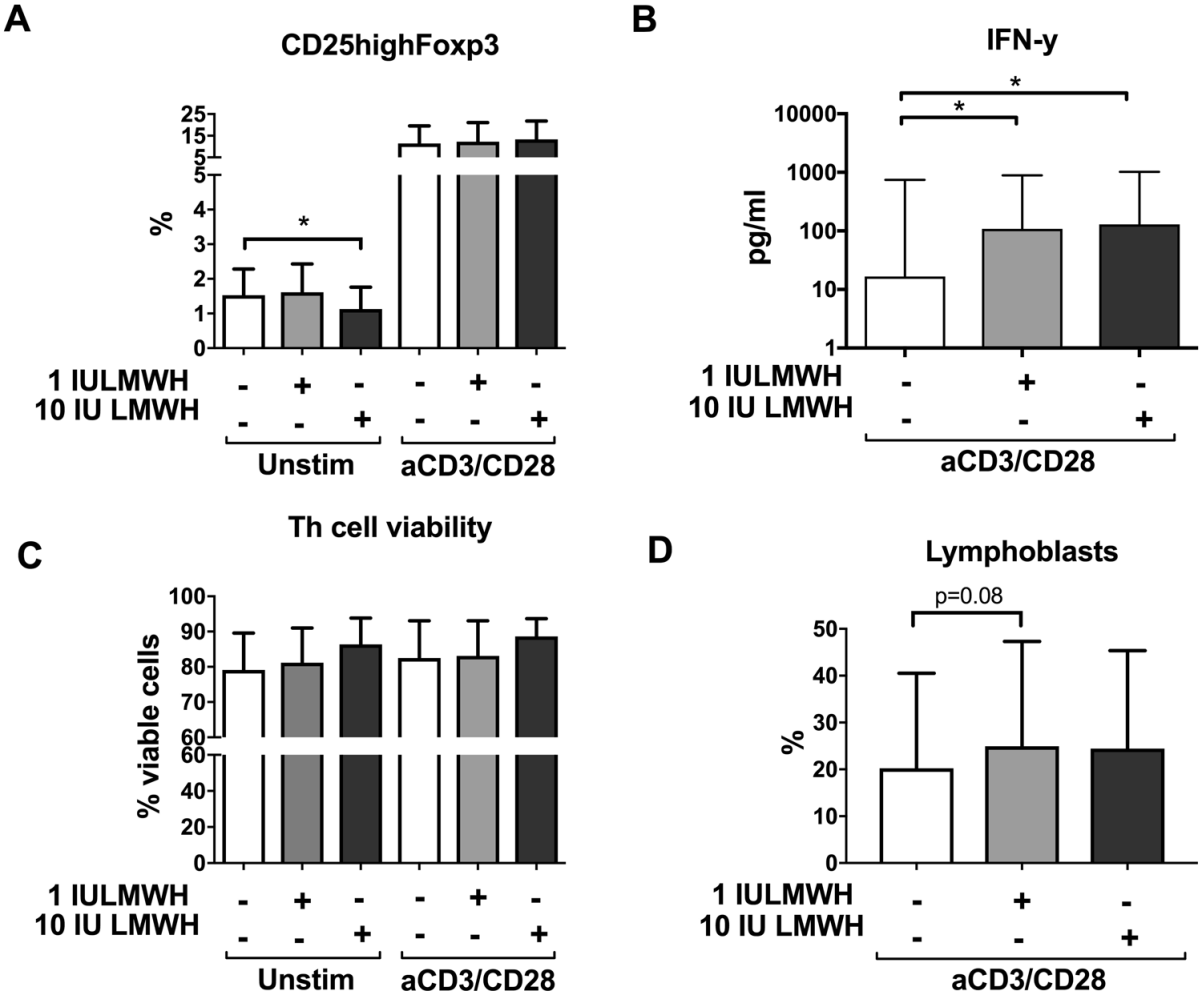
Effects of LMWH on Th cells. Effects of LMWH on Th cell phenotype (**A**), cytokine secretion (**B**) and viability and activation (**C**,**D**), measured as proportion (%) of viable cells and of lymphoblasts. CD4^+^ T cells, both unstimulated and stimulated with anti-CD3 and anti-CD28 Abs, were cultured for 3 days in the absence or presence of 1 or 10 IU LMWH. Data are expressed as mean ± S.D. (**A**,**C**,**D**) and as box plots (**B**) showing interquartile range (box) and range (whiskers). One way ANOVA and Sidak’ s test: *p < 0.05 (**A**). Friedman and Wilcoxon tests (**B**): *p < 0.05. One way ANOVA and Sidak’ s test (**C**,**D**); (**A**) n = 7, (**B**) n = 19 (un-exposed) and n = 17 (exposed to LMWH), (**C**) n = 18 (un-exposed and exposed to 1 IU LMWH in T cell stimulated samples), n = 13 (exposed to 1 IU LMWH in T cell unstimulated samples), n = 10 (exposed to 10 IU LMWH in T cell unstimulated samples), n = 15 (exposed to 10 IU LMWH in T cell stimulated samples), (**D**) n = 15.

To assess the effects of LMWH on Th cells, we cultured CD4^+^ T cells from healthy non-pregnant women in the presence or absence of LMWH. To mimic a “resting state” microenvironment, CD4^+^ T cells were cultured in the absence of T cell receptor (TCR)-stimulation (“unstimulated”). Unstimulated CD4^+^ T cells exposed to 10 IU of LMWH showed a decreased proportion of CD25^high^Foxp3^+^ Treg cells (20% decrease) (Fig. 3A, a representative dot plot is shown in Supplemental Fig. 1G,H). This decrease was in line with enhanced secretion of IFN-γ (554–678% increase), which was noted in anti-CD3/CD28-stimulated Th cells (*i.e*. representing a pro-inflammatory environment) upon LMWH exposure (Fig. 3B). Conversely, the proportions of T-bet^+^, GATA-3^+^ and Roryt^+^ Th cells (Th1, Th2 and Th17, respectively) were not significantly affected by LMWH (Supplemental Fig. 4) and the production of GM-CSF, IL-2, IL-10, IL-17, IL-1β, IL-6, and TNF by Th cells was not altered after exposure to LMWH (Supplemental Fig. 5). Furthermore, LMWH exposure tended to increase (by 23%) the proportion of lymphoblasts (activated T cells) in anti-CD3/CD28-stimulated Th cell cultures compared to the non-exposed ones (p = 0.08 in the ANOVA, p < 0.05 in the post hoc test) (Fig. 3D). The proportions of the activation markers HLA-DR, CD25 and CD69 on Th cells were not significantly affected by LMWH treatment (Supplemental Fig. 6).

### Effects of LMWH on placenta explants

**Figure 4:**
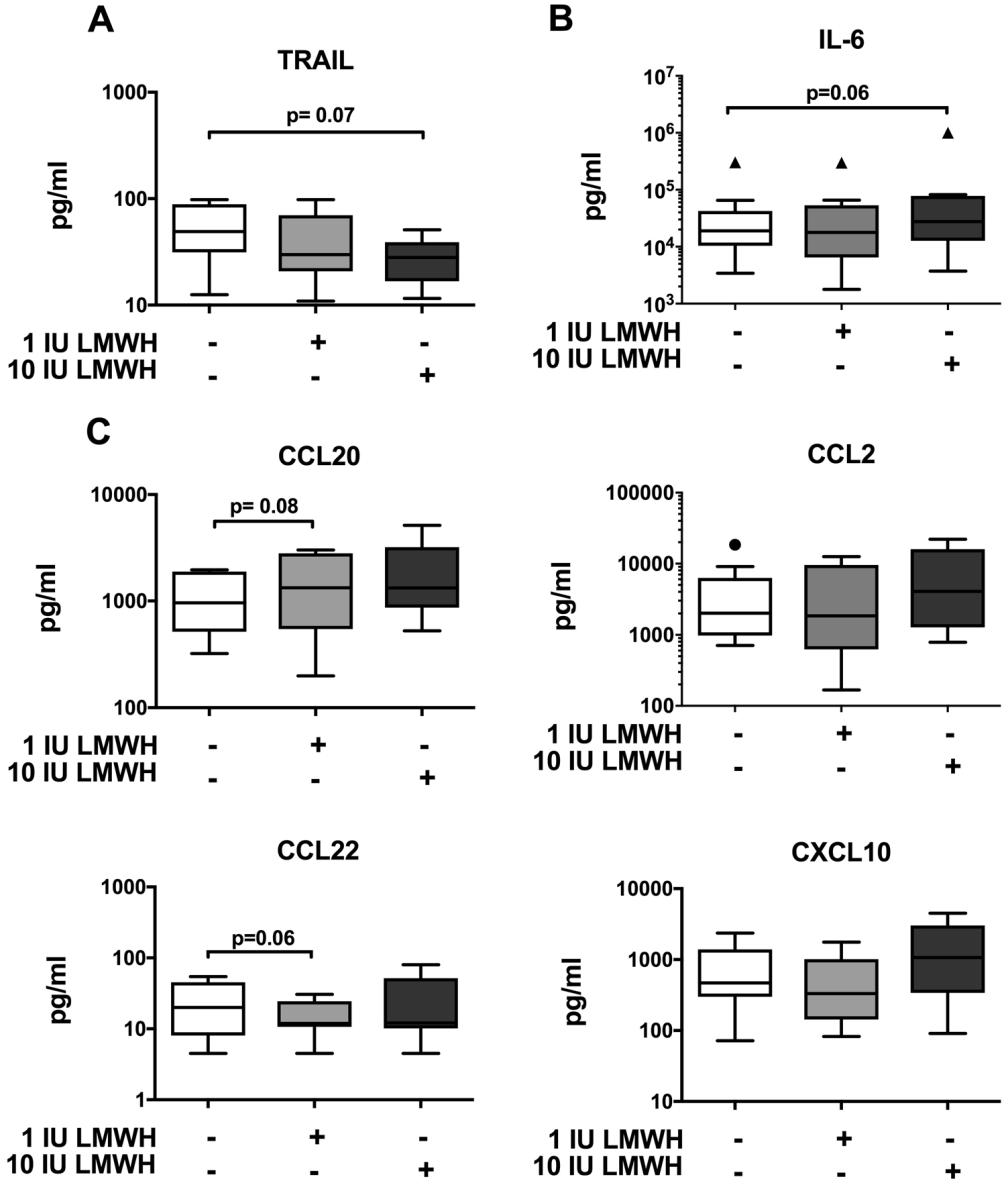
Effects of LMWH on 1^st^ trimester placental explants. Effects of LMWH on the production of soluble factors by placental explants. Placenta explants were exposed or not to 1 or 10 IU of LMWH for 20–24 hours. Data are expressed as median and inter-quartile range (box) and range (whiskers). Friedman test followed by Wilcoxon; n = 10.

A wide panel of soluble factors were analysed in CM obtained from healthy 1^st^ trimester placenta explants not exposed or exposed to different doses of LMWH (1 IU and 10 IU). LMWH exposure tended to decrease (by 43%) the secretion of the pro-apoptotic molecule TRAIL compared with the non-exposed tissue (p = 0.07 in the Friedman test, p < 0.05 in the Wilcoxon test) (Fig. 4A). There were also tendencies towards decreased secretion of CCL22 (p = 0.06) (Fig. 4C) and increased secretion of IL-6 (p = 0.06) (Fig. 4B) and CCL20 (p = 0.08) (Fig. 4C) from placental explants exposed to LMWH. LMWH did not affect CXCL 1–3, CXCL8, M-CSF, GM-CSF, IL-1β or IL-10 secretions in placental explants (Supplemental Fig. 7).

## Discussion

In the present study, we examined *in vitro* effects of LMWH on macrophages and Th cells, two key players in promoting immune tolerance during pregnancy. By mapping of phenotypic changes as well as induction of cytokine and chemokine secretion, we show mainly activating pro-inflammatory effects of LMWH on both macrophages and Th cells.

Regarding macrophages, the assumption of LMWH-induced activation was based on the increased expression of the classical activation marker HLA-DR. Furthermore, LMWH also increased the expression of CD206, which has been considered being an M2 marker. However, the expression of CD206 increases in the presence of GM-CSF^25^, which induces a pro-inflammatory condition^25^, and therefore, to some extent, reflects general activation. In further support of this notion, LMWH exposure did not increase expression of the major decidual macrophage marker CD163. While these phenotypic changes were statistically significant, they had a rather small magnitude. Changes in chemokine secretion, on the other hand, were pronounced after LMWH exposure, with a decreased secretion of CCL2. CCL2 is expressed and secreted by decidual macrophages^24,25^, and has been associated with recruitment of Th2 cells^31^, as well as reduced production of proinflammatory cytokines by macrophages^32^, thus indicating that the decrease in CCL2 secretion is in line with a LMWH-induced pro-inflammatory profile of macrophages. In addition, the chemokine profile of macrophages also reflects polarized immune responses that are associated with different Th subsets. In this respect, LMWH exposure decreased macrophages’ secretion of Th2-associated CCL22, while it increased Th17-associated CCL20. Although the role of Th17 in pregnancy is not settled, it is known as a mainly proinflammatory cytokine^33^, and Th2 is known to promote successful pregnancy^33^. Thus, an increase in CCL20 and a decrease in CCL2 and CCL22, together with the changes in phenotype, indicate that macrophages exposed to LMWH over all induced a mainly activated pro-inflammatory profile *in vitro*. In line with our finding of a direct LMWH effect on monocyte to macrophage differentiation, it has been reported that LMWH can bind to the cell surface of monocytes^14,15^. Further, our finding of an inflammatory profile is in accordance with reports of heparin´s enhancement of LPS-induced secretion of the proinflammatory cytokines CXCL8 and IL-1β by mononuclear cells and monocytes^16,17^. However, other studies showed a reduced monocyte secretion of proinflammatory cytokines IL-1β, IL-6 and TNF in the presence of heparin^18–20^. Of note, our study is the first to evaluate LMWH effects on the macrophage differentiation and polarization process. Other causes for discrepant results include differences in study design (length and dose of stimulation, isolated or mix of cells, stimulatory conditions, type of heparin, and read-out), choice of donors (sex, age, treatment), and size of the study.

LMWH affected Th cells in a consistent way; a decrease in the proportion Treg cells (noted in the resting condition) and an increase in secretion of Th1-associated IFN-γ (noted in the activated condition), as well as a tendency to an increased proportion of lymphoblasts, thus in line with an activating and mainly pro-inflammatory effect of LMWH, as was the case for macrophages. In the present study, we used a stringent statistical approach, implying that differences that were statistically significant showed an increase or decrease in the order of 17 to 678% relative to unexposed cells, being more pronounced in the functional assay of cytokine/chemokine secretion than in the phenotypic assessments. Although it remains to be settled whether the magnitude of changes would be of biological significance, the findings were in the same direction, in favour of a biological relevance. To the best of our knowledge, our finding is the first documentation of direct effects of LMWH on Th cells *in vitro*. It was previously shown *in vivo* that LMWH treatment may increase Treg cell frequency in peripheral blood^34^. This finding shows that *in vivo* may differ from *in vitro*, one parameter being that we examined direct effects while *in vivo* involves several other cellular and molecular interactions including the possibility of LMWH effects on cell traffic, which has been shown in the context of innate immunity^13^. It is therefore important to consider that the increased Treg frequency noted in blood may not be paralleled by an increase at the fetal-maternal interface^23^. This is a crucial point for RM treatment, since immune modulation is particularly needed at the maternal-fetal interface and involves Treg cells and regulatory M2 macrophages as key players in immune tolerance^35^, processes that are inadequate in RM^2^.

In addition to LMWH effects on macrophages and Th cells, we also evaluated LMWH effects on the placenta, since the placenta is able to induce tolerance promoting effects on macrophages and Th cells^35^. Thus, LMWH could indirectly affect these cells by affecting the placenta. Although minor changes were found regarding cytokines and chemokines, the tendencies observed with a reduction in CCL22 (Th2) and an increase in CCL20 (Th17) and IL-6, are in line with the direct effects on macrophages, and suggestive of a mainly pro-inflammatory role of LMWH. Furthermore, LMWH tended to reduce TRAIL secretion from the placental explants, supporting a role of LMWH in promoting cell survival at maternal-fetal interface^10^. TRAIL has been localized mainly to the syncytiotrophoblast within the placenta, and has been ascribed a beneficial role in fetal immune tolerance^36^. In mice, TRAIL was shown to preferentially expand the Treg population and to inhibit expansion of non-Treg cells^37^. In accordance, we recently showed that blocking of TRAIL led to a significant reduction in the proportion of Foxp3^+^ Treg cells induced by human placental explants^22^. Thus, a LMWH-induced reduction of TRAIL secretion from placental explants could have negative effects for fetal tolerance.

A mainly pro-inflammatory effect on macrophages and Th cells, two central tolerance-promoting cells at the maternal-fetal interface, may suggest the possibility of a present non-optimal use of LMWH in RM, when administered for its immunological and not its anti-coagulant effects. RM is characterized by a proinflammatory profile, including increased numbers of Th17^29,33,38^ and Th1 cells^33^, and reduced numbers of IL-10 secreting macrophages^28^. Therefore, the possible pro-inflammatory effects of LMWH as shown in the present study, could rather suggest a role for LMWH in the earliest phases of pregnancy, especially during implantation, when a mainly proinflammatory process is required. In fact, it has been hypothesized that an inadequate implantation process, with a weak pro-inflammatory response, could be a contributing factor to RM. An early LMWH treatment is also supported by its well-documented effect on prevention of trophoblast apoptosis^10^, enhancement of trophoblast invasiveness^11^, improvement of the endothelial and vascular environments, and regulation of embryo implantation^12^. Furthermore, LMWH has been shown to modulate the decidualization of endometrial stromal cells (ESCs) *in vitro* by improving endometrial receptivity and supporting early implantation^39^. These processes have been shown to be abnormal in women affected by RM, with a consequent extension of the implantation window and an increased ability of the decidualized endometrium to allow implantation of poor quality embryo (endometrial super-receptivity)^40^. Therefore, although speculatively, LMWH could even be administered during the luteal phase, and be beneficial for a proper implantation, which in turn might decrease the risk for RM. In fact, it has been demonstrated that LMWH administration from the luteal phase could improve the implantation and live birth rates in women with repeated implantation failure undergoing assisted reproduction techniques^41^.

The contradicting findings in LMWH clinical trials to date could in part be explained by the attempt to put together non-homogenous data, since, hypothetically, an early start of LMWH treatment could be beneficial, while treatment at later gestational ages might have negative effects. Since a majority of studies have not been designed to test the hypothesis about the beneficial effects of LMWH if administrated at the time of initial implantation^42^, further studies are clearly needed to confirm this hypothesis.

## Conclusion

Here we show that LMWH induces a mainly pro-inflammatory profile on two central tolerance-promoting cells at the maternal-fetal interface. Although the biological significance of these *in vitro* findings should be verified *in vivo*, together with previously reported anti-apoptotic and invasion-promoting LMWH effects on trophoblasts, they could suggest the possibility of using LMWH at an earlier gestational age in women with RM, where the anticoagulant effect is not the primary goal of treatment.

## Electronic supplementary material

Supplemental Data
