# Supplementary material for: Effects of low molecular weight heparin on the polarization and cytokine profile of macrophages and T helper cells in vitro
Source: Sci Rep. 2018 Mar 8;8:4166. doi: 10.1038/s41598-018-22418-2 (PMC5843640; doi:10.1038/s41598-018-22418-2)
Supplement: Supplementary file 1 — Supplemental Data [file 41598_2018_22418_MOESM1_ESM.pdf]

Effects of low molecular weight heparin on the polarization and cytokine profile of macrophages and T helper cells *in vitro*

***Bruno Valentina [a,b], *Svensson-Arvelund Judit [b], Rubér Marie [c], Berg Göran [c], Piccione Emilio [a], Jenmalm Maria C. [b], Ernerudh Jan [d]**

* These authors contributed equally to the paper

[a] Academic Department of Biomedicine and Prevention, Section of Gynecology and Obstetrics, University of Rome Tor Vergata and Department of Surgery, Section of Gynecology and Obstetrics, Tor Vergata University Hospital, Viale Oxford, 81, Rome, Italy

[b] Department of Clinical and Experimental Medicine, Linköping University, SE-581 83 Linköping, Sweden

[c]Department of Obstetrics and Gynecology, and Department of Cinical and Experimental Medicine, Linköping University, SE-581 83 Linköping, Sweden

[d]Department of Clinical Immunology and Transfusion Medicine, and Department of Clinical and Experimental Medicine, Linköping University, SE-581 83 Linköping, Sweden

Correspondence to: Dr. Valentina Bruno, M.D., Department of Biomedicine and Prevention, Section of Gynecology and Obstetrics, University of Rome Tor Vergata and Department of Surgery, Section of Gynecology and Obstetrics, Tor Vergata University Hospital, Viale Oxford, 81, 00133, Rome, Italy,

tel. +39 0620902924, e-mail: valentinabruno_86@hotmail.it

Supplemental data.

Supplemental table I. Antibodies used for flow cytometry.

Cell type	Antigen	Isotype	Fluorochrome	Manufacturer
**Macrophages**	HLA-DR	Mouse IgG2b	FITC	BD Biosciences
	CD 163	Mouse IgG1	PE	BD Biosciences
	CD 206	Mouse IgG1	APC	BD Biosciences
	CD 209	Mouse IgG2b	PerCP-Cy5.5	BD Biosciences
	CD86	Mouse IgG1	V450	BD Biosciences
**T helper cells**	CD4	/	FITC	BD Biosciences
	CD25	Rat IgG2a	PC-7	BioLegend
	GATA-3	Mouse IgG2b	PerCP-Cy5.5	BioLegend
	Foxp3	Rat IgG2a	PE	eBioscience
	Rorγt	Rat IgG2a	APC	eBioscience
	T-bet	Mouse IgG1	Pacific Blue	BioLegend

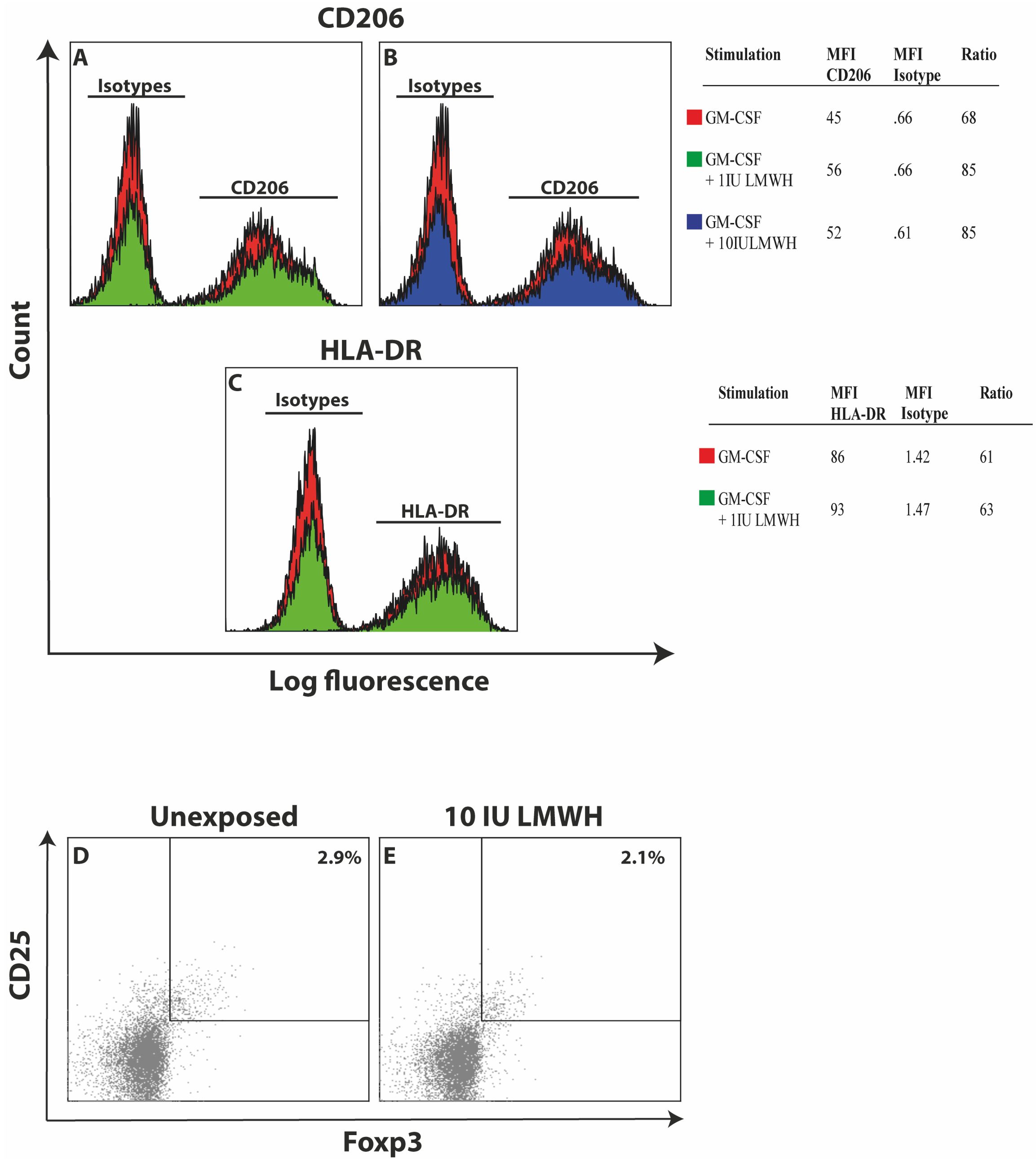

Stimulation	MFI CD206	MFI Isotype	Ratio
GM-CSF	45	.66	68
GM-CSF + 1IU LMWH	56	.66	85
GM-CSF + 10IULMWH	52	.61	85

Stimulation	MFI HLA-DR	MFI Isotype	Ratio
GM-CSF	86	1.42	61
GM-CSF + 1IU LMWH	93	1.47	63

Supplemental Figure 1. Representative examples of macrophage (1A-C) and T cell (1D-E) markers, measured as fluorescence intensity (1A-C) or proportion of cells (%, 1D-E). Blood monocytes were cultured for 6 days with GM-CSF (red colour in histograms) in the absence or presence of 1 IU (green colour in histograms) or 10 IU (blue colour in histograms) LMWH. Isolated blood CD4 T cells were cultured for 3 days in the absence or presence of 10 IU LMWH. A and B show an increase in median fluorescence intensity (MFI) of CD206 when exposed to 1 IU (MFI, 56) or 10 IU (MFI, 52) LMWH as compared with LMWH unexposed cells (MFI, 45). C shows an increase in median fluorescence intensity (MFI) of HLA-DR when exposed to 1 IU (MFI, 93) LMWH as compared with the LMWH unexposed cells (MFI, 86). The MFIs of CD206, HLA-DR and their corresponding isotype controls, as well as ratios (CD206 or HLA-DR MFI/corresponding isotype controls MFIs) are shown in the right panels. D and E are representative examples showing increased proportions of CD25highFoxp3+ Treg cells in the presence of 10 IU LMWH compared with unexposed cells.

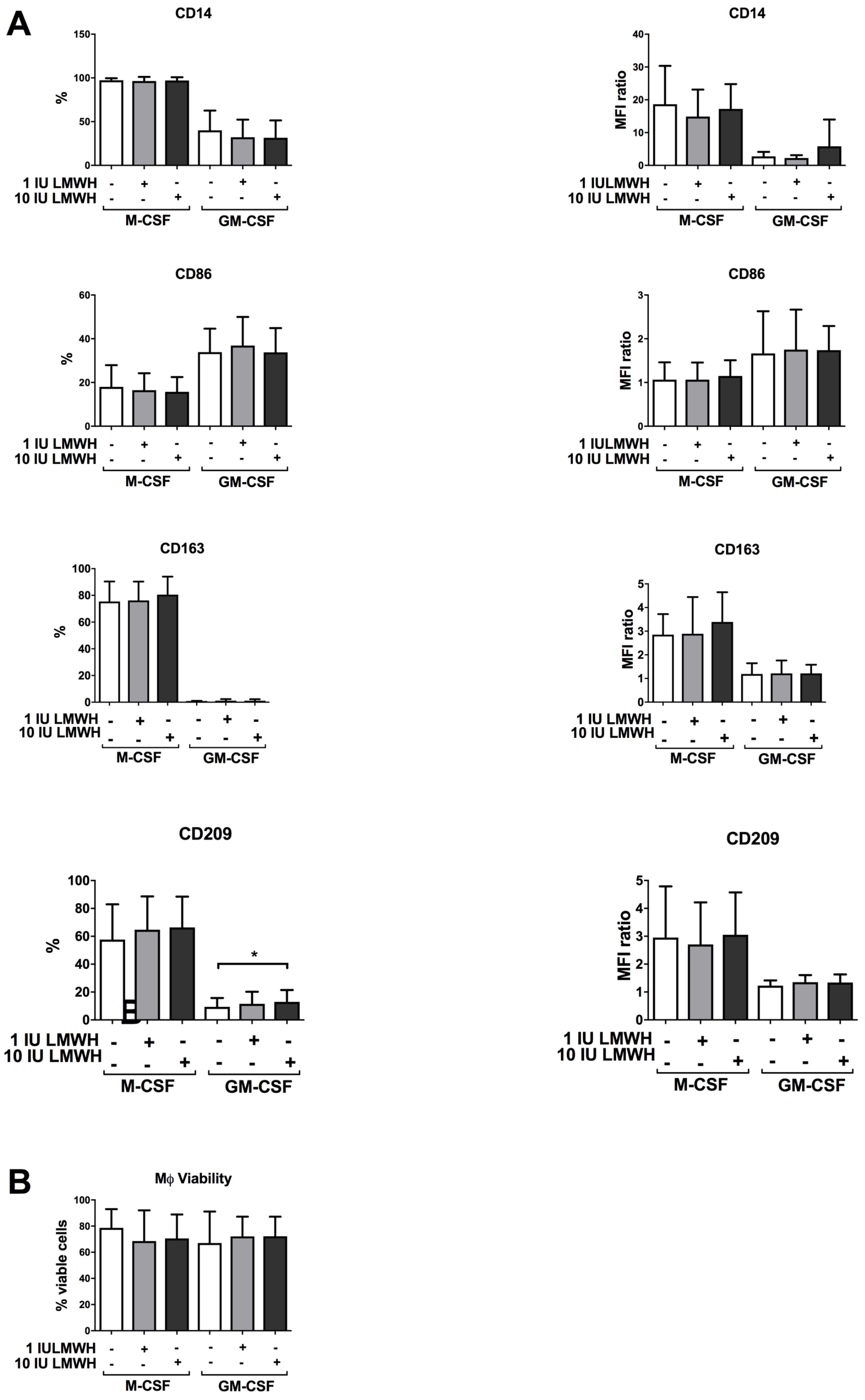

Supplemental Figure 2. Effects of LMWH on macrophage polarization and viability.

Effect of LMWH on macrophage phenotype markers, measured as proportion of cells (%) and relative expression of the entire population (median fluorescence intensity, MFI) (A) and on viability (B). Macrophages were cultured with GM-CSF and M-CSF in the absence or presence of 1 or 10 IU LMWH. One Way ANOVA (A) and Student' s t- test (B) were performed: no statistical significant differences were found; (A) n = 8, (B) n = 7.

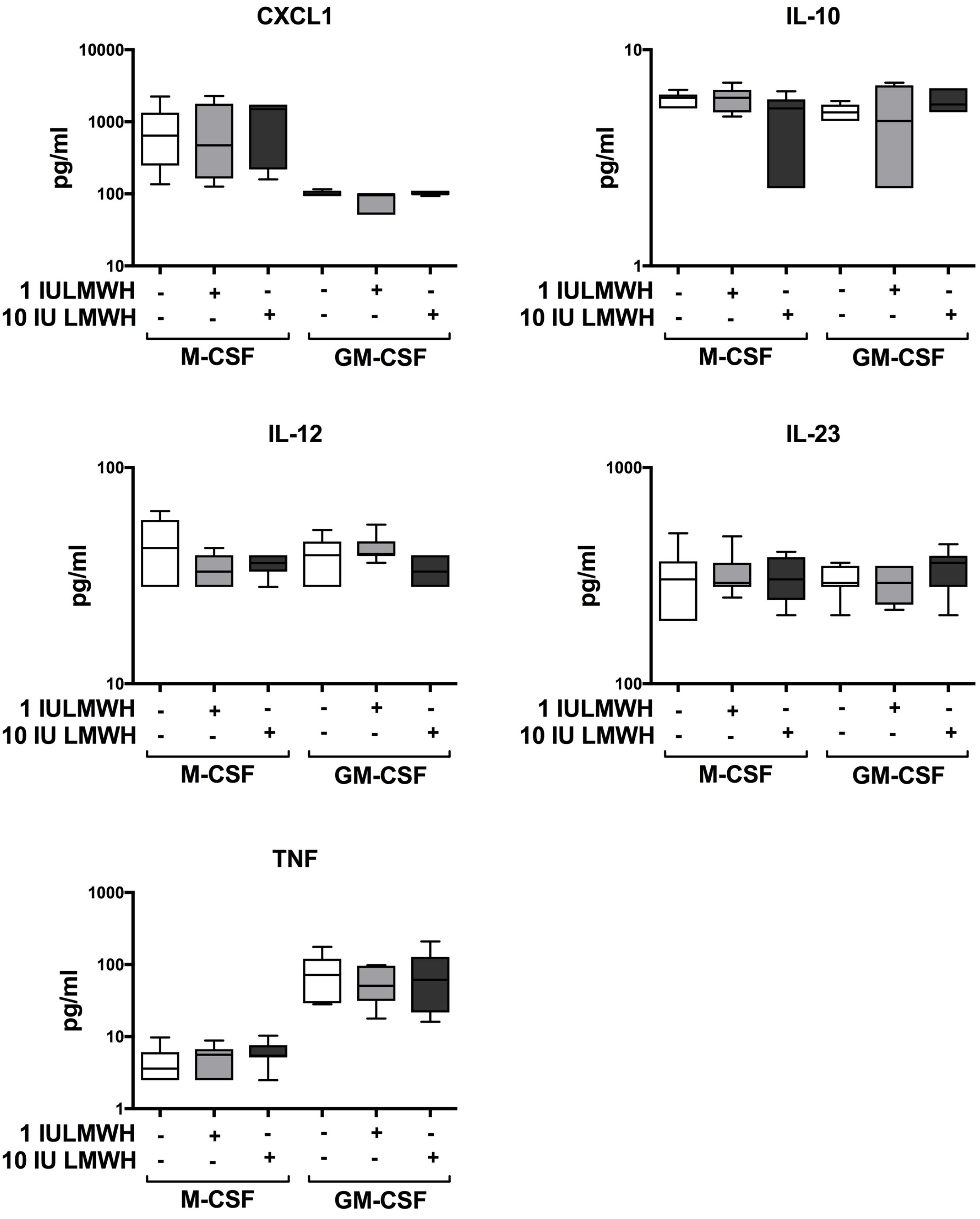

Supplemental Figure 3. Effects of LMWH on the production of chemokines and cytokines by macrophages.

Macrophages were cultured with GM-CSF and M-CSF in the absence or presence of 1 or 10 IU LMWH. Friedman test was performed: no statistical significant differences were found; n = 7.

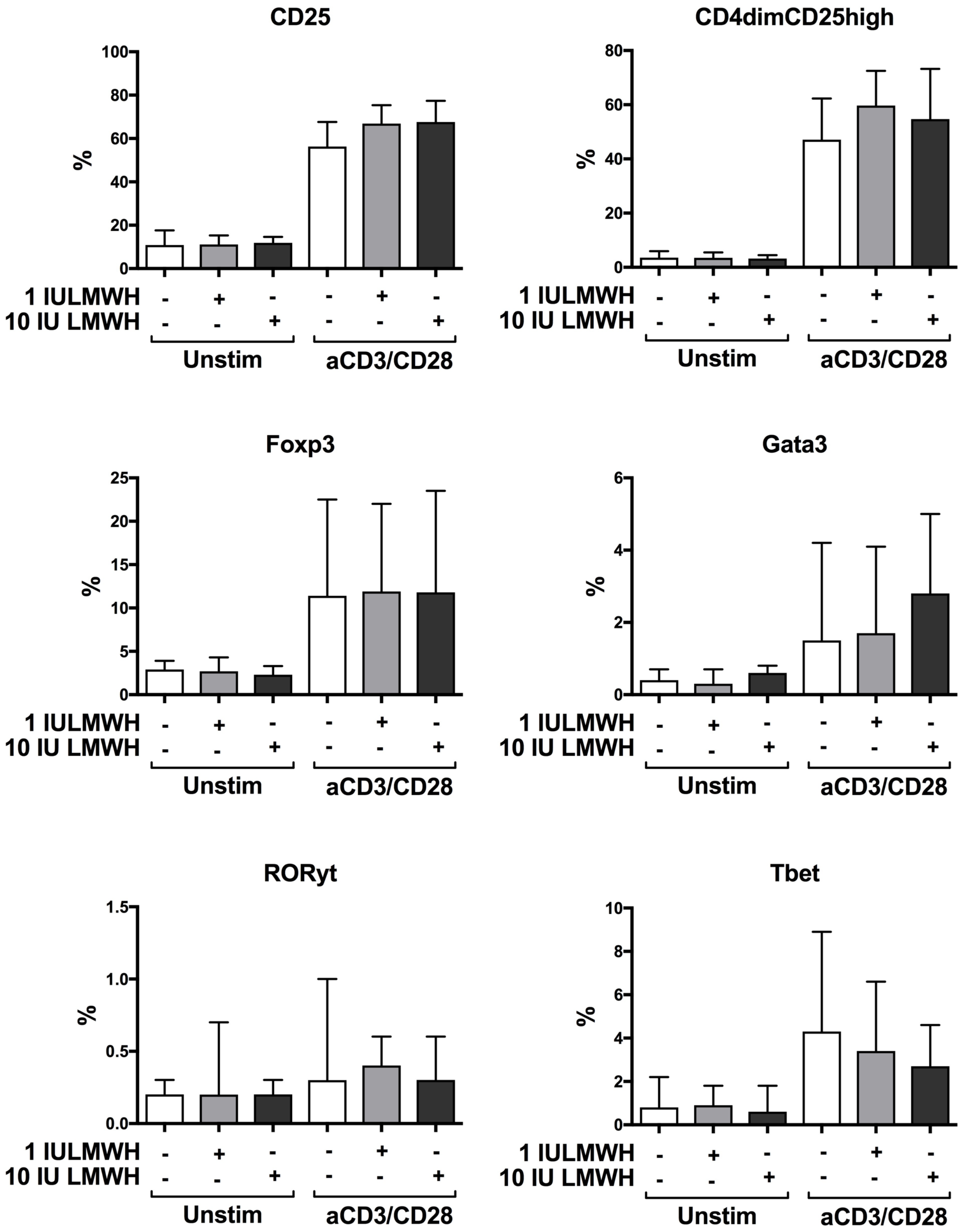

Supplemental Figure 4. Effects of LMWH on Th cells polarization.

Effects of LMWH on Th cell phenotype. $CD4^+$ T cells, both unstimulated and stimulated with anti-CD3 and anti-CD28 Abs, were cultured in the absence or presence of of 1 or 10 IU LMWH. One Way ANOVA was performed: no statistical significant differences were found; n = 7.

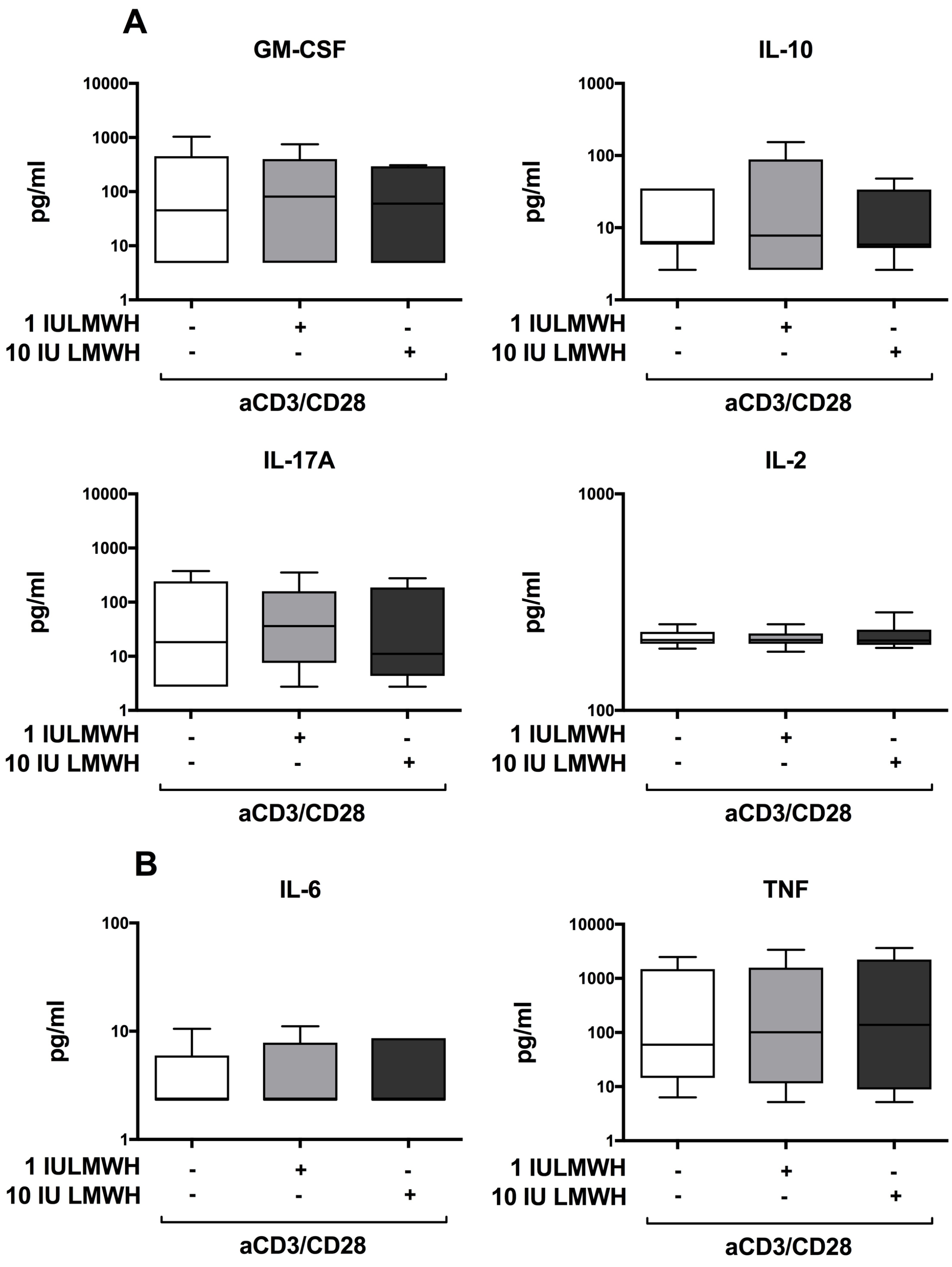

Supplemental Figure 5. Effects of LMWH on the production of cytokines and chemokines by Th cells.

CD4⁺ T cells, both unstimulated and stimulated with anti-CD3 and anti-CD28 Abs, were cultured in the absence or presence of 1 or 10 IU LMWH. Friedman test was performed: no statistical significant differences were found. IL-1β was undetectable (data not shown). (A) n = 19 (unexposed and exposed to 1 IU LMWH) and n = 17 (exposed to 10 IU LMWH), (B) n = 11.

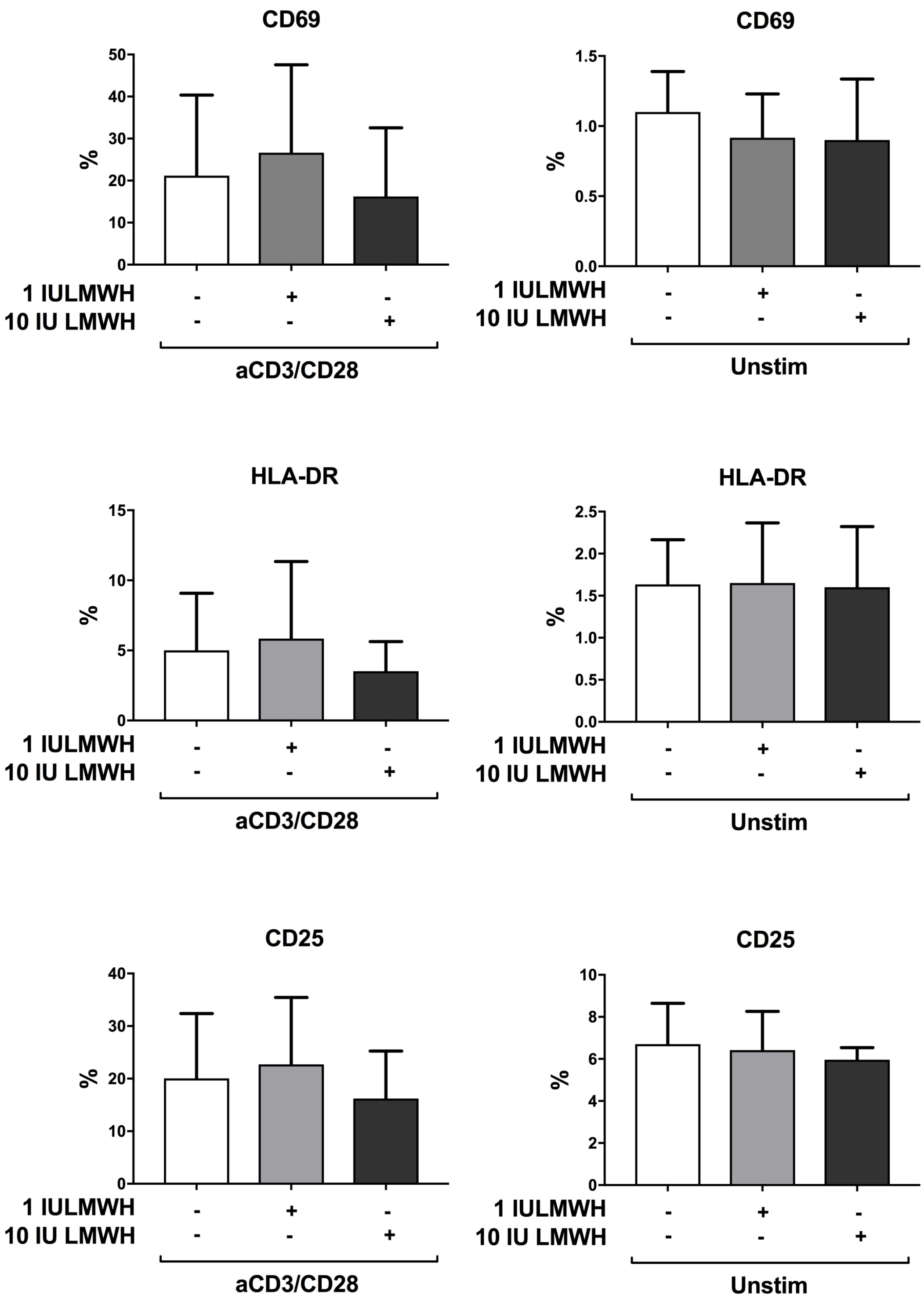

Supplemental Figure 6. Effects of LMWH on Th cells activation markers.

Effects of LMWH on Th cell activation markers. $CD4^+$ T cells, both unstimulated and stimulated with anti-CD3 and anti-CD28 Abs, were cultured in the absence or presence of of 1 or 10 IU LMWH. One Way ANOVA was performed: no statistical significant differences were found; n = 11 (unexposed and exposed to 1 IU LMWH in T cell stimulated samples), n = 8 (exposed to 10 IU LMWH in T cell stimulated samples), n = 6 (unexposed and exposed to 1 IU LMWH in T cell unstimulated samples), and n = 3 (exposed to 10 IU LMWH in T cell unstimulated samples).

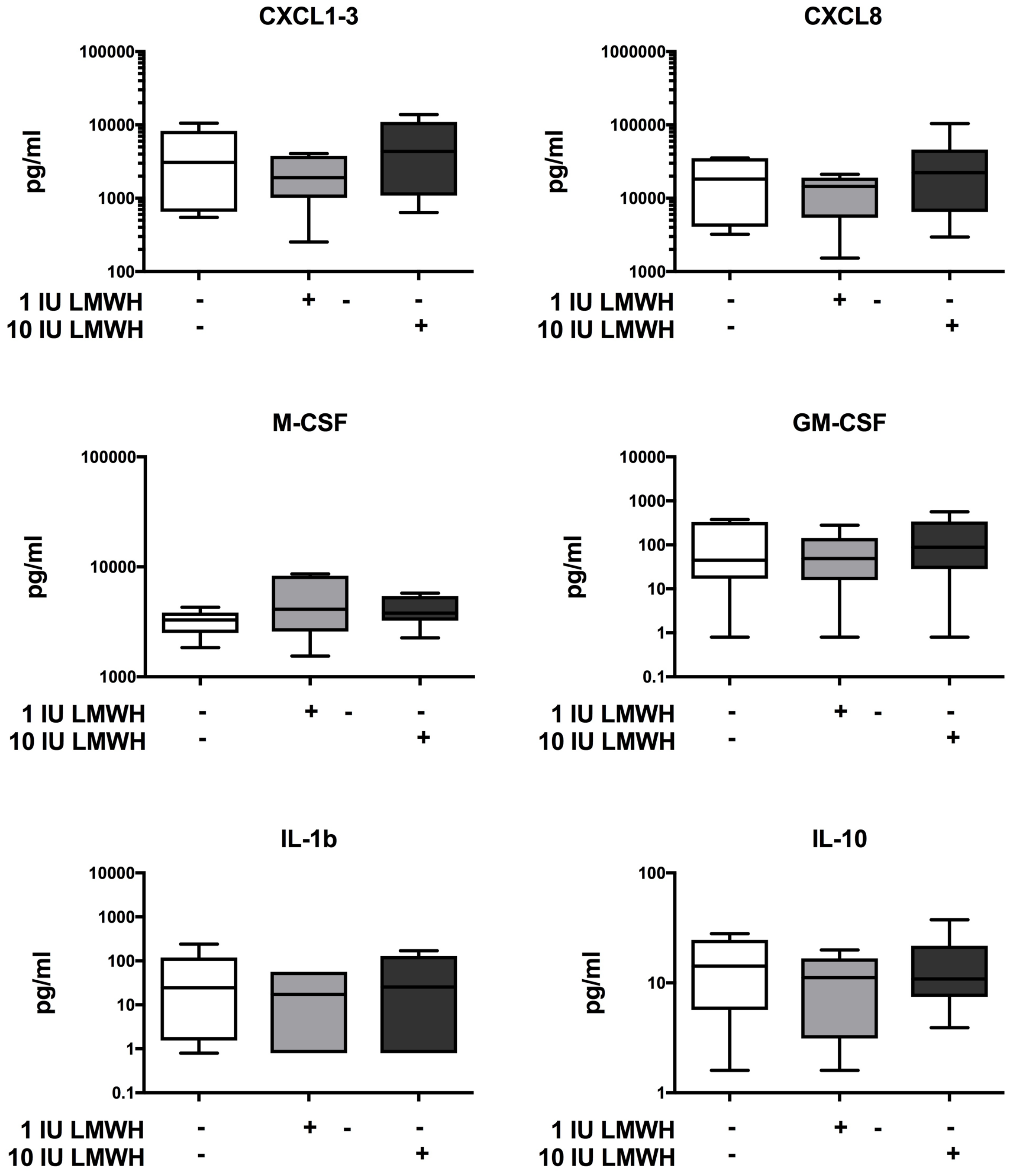

Supplemental Figure 7. Effects of LMWH on 1st trimester placental explants.

Effects of LMWH on the production of soluble factors by placental explants. Placenta explants were exposed or not to 1 or 10 IU of LMWH. Friedman test was performed: no statistical significant differences were found. VEGF and sFasL were undetectable (data not shown). n = 10.
